# The Oral Mucosa Status and the Correlation between the Functional Parameters and the Level of Nitric Oxide Metabolites in Saliva among Patients with GERD

**DOI:** 10.1155/2020/1273031

**Published:** 2020-11-12

**Authors:** G. I. Lukina, A. V. Ivannikova, M. Y. Abramova, E. M. Kuzmina, A. V. Lukin, A. V. Alimova, A. B. Slabkovskaya

**Affiliations:** ^1^A. I. Yevdokimov Moscow State University of Medicine and Dentistry, Moscow, Russia; ^2^State Autonomous Healthcare Institution “Dental Clinic No 8 of Moscow Health Department”, Moscow, Russia

## Abstract

The study involved 91 patients (48 women and 43 men), aged from 18 to 70 years with GERD. All patients underwent the clinical dental examination according to a single scheme including general clinical manifestations (nausea, single vomiting, belching, heartburn, pain in the epigastrium and around the navel, and poor appetite) and dental manifestations of GERD. The objective assessment of the dental status of the examined patients included the measurement of the functional parameters of the mixed saliva, buffer capacity (BC) of saliva, and the detection of the nitric oxide metabolites (NOx) content in saliva from the right parotid salivary gland (“SRPSG”) and in blood serum using the indirect method based on the determination of the stable metabolites: nitrates and nitrites using the Griess reaction. It was established that salivation rate among patients with GERD with the prevailing of ACR and SACR was at the lower limit of normal values (0.32 + 0.19 ml/min), and the salivation rate among patients with the prevailing of SALCR was low (0.10 + 0.04 ml/min). The BC of saliva among patients with the prevailing of ACR and SACR was high (9.07 + 1.23 mmol eq/l and 9.40 + 1.71 mmol eq/l, respectively) and was reduced among patients with the prevailing of SALCR (7.63 + 0.18 mmol eq/l). The NOx level in SRPSG among patients with GERD was increased (especially in Group 3 (20.93 + 11.23 umol/l)). The direct correlation between the indicators of sialometry, the level of the BC of saliva, and the NOx level in SRPSG were established during the study.

## 1. Introduction

Gastroesophageal reflux disease (GERD) is extremely common today and covers about 40.0% of developed countries' adult population; in Eastern Europe countries, it occurs in 40.0% to 60.0% of the population, and among those patients with GERD, a rather large proportion of patients (45.0–60%) has the extraesophageal sort of GERD manifestations including a wide range of dental pathology [1, 2, 4, 5. 6, 8].

According to the modern ideas, the formation of GERD is associated with the action of many factors which determine the weakness of the lower esophageal sphincter. The leading one is the nitric oxide [1]. The increase of its concentration leads to decrease of the lower esophageal sphincter tone and, in turn, contributes to the development of the pathological gastroesophageal reflux. The level of intracellular calcium (which in turn contributes to the connection of actin and myosin filaments and thus provides a muscle fiber reduction) decreases while binding nitric oxide with guanylate cyclase in muscle layers, and the lower esophageal sphincter respectively relaxes. The decrease of the antireflux barrier can lead to the flowing back of the stomach content or the stomach and the duodenal contents with the subsequent connection of the high reflux components to the oral cavity. Based on the above, the determination of nitric oxide level and, accordingly, its metabolites in the biological fluids play an important role in the pathogenesis of GERD and the dental pathology on the background disease [2].

The purpose of this study is to determine the clinical status, the dependence of the saliva functional parameters from the level of nitric oxide metabolites from the parotid salivary gland among patients with GERD with a different pH of refluctant.

## 2. Materials and Methods

### 2.1. The Objectives of the Study

1. To conduct a retrospective analysis of the results of 24-hour intraesophageal pH-impedancemetry and clinical examination of patients with GERD depending on the predominant nature of the refluctate.
2. To study the clinical condition of oral organs in patients with GERD depending on the predominant nature of the reflux.
3. To measure the quantitative and qualitative parameters of mixed saliva (salivation rate, pH-metry, and buffer capacity of saliva).
4. To examine the pathogenetic role and features of the metabolism of nitric oxide nitrogen in patients with GERD depending on the dominant character of refluctant.

In total, 230 patients were examined at the dental appointments. From the anamnesis, it was found that 53 patients suffered from GERD of varying severity, which was observed by a gastroenterologist. For 63 patients, according to the manifestations in the oral cavity (a feeling of bitterness in the mouth, the presence of heartburn, dry mouth, profuse salivation, and lining of the tongue), it was decided to send them for consultation and clinical examination to a gastroenterologist. In 30 patients, the diagnosis of GERD was confirmed, and they were invited to participate in the study. 9 patients with GERD were referred by a gastroenterologist for consultation and treatment to a dentist.

Therefore, the study involved 91 patients (48 women and 43 men), aged from 18 to 70 years. The study was approved by the Intercollegiate Ethics Committee. The study was conducted on the basis of MSMSU named after A. I. Yevdokimov, SAHI “Dental clinic no. 8 of Moscow Health Department,” in the laboratory of cell biotechnology, as well as in the laboratory of functional diagnostics of gastroenterological diseases, functioning on the basis of the clinic for propedeutics of internal diseases, gastroenterology, and hepatology of the UKB no. 2. Every patient participating in the study gave an informed consent and signed an informed consent form.

All the participants of the study were divided into the control group (25 patients seeking dental assistance but without the manifestation of background pathology, in which the number of daily and high refluxes did not go beyond the normal range) and 3 main groups, according to the retrospective analysis of the 24-hour intraesophageal pH-impedance results. In main groups (*n* = 67), patients with GERD with a high gastroesophageal reflux (which was above 17 cm from the bottom of the esophagus) were recruited. The division was dependent from the pH in the lumen of the esophagus: Group 1, patients with GERD with the prevailing of the acidic character of refluctant (ACR) (pH in the esophagus was <4) (*n* = 25); Group 2, patients with GERD with the prevailing of the slightly acidic character of refluctant (SACR) (4 < pH < 7) (*n* = 25), and Group 3, patients with GERD with the prevailing of the slightly alkaline character of refluctant (SALCR) (pH < 7) (*n* = 17).

All patients underwent the clinical dental examination according to a single scheme: the clarification of complaints through the use of questionnaires, anamnesis, external examination, and the examination of the oral cavity. Particular attention was paid to general clinical manifestations (nausea, single vomiting, belching, heartburn, pain in the epigastrium and around the navel, and poor appetite) and dental manifestations of GERD, excluding manifestations on hard tissues of teeth.

The following complaints were considered in a subjective assessment of dental manifestations of GERD: the complaints of bitterness in the mouth, feeling of sour in the mouth, halitosis, plentiful plaque on the tongue, xerostomia, hypersalivation, and discomfort in the mouth.

The objective assessment of the dental status of the examined patients included the following:

(1) the measurement of the functional parameters of the mixed saliva (the determination of the salivation rate by the method of T. Redinova and R. Pozdeev (1994). For this study, the following equipment was used: a stopwatch, a graduated tube, and a funnel. The patient was asked to tilt his head to the chin, to sit in this position, not to talk, not to swallow saliva, and not to move his lips and tongue for 2 minutes. Then, the patient was asked to spit the accumulated saliva into a funnel placed in a graduated tube. The operation was repeated 2 more times for a total of 6 minutes. The salivation rate was calculated using the formula:

Cc = *V*/*t*, where “Cc” is the rate of salivation, “*V*” is the volume of saliva released (ml), and “*t*” is the time of saliva collection (in minutes).

(2) The buffer capacity (“BС”) of saliva by the method of K. Leontiev (1974): first, the pH of the mixed saliva is measured, and then 1.0 ml of 0.01 N hydrochloric acid solution is added to 1.0 ml of saliva and the pH is determined again. Next, 1.0 ml of 0.01 N NaOH solution is added to the same volume of saliva and the pH is determined again. The BC is calculated by the formula:

BC = 10/(pH_0_ − pH_1_) *x* B, where BC is the buffer capacity of 1.0 l of saliva, in meq. acids or alkalis; 10 is the conversion factor per 1.0 l of saliva; (pH_0_ − pH_1_) is the difference in pH units before and after adding 1.0 ml of 0.01 N acid or alkali solution; B is the volume of saliva taken for analysis.

(3) The detection of the nitric oxide metabolites (NOx) content in saliva from the right parotid salivary gland (SRPSG) and in blood serum using the indirect method based on the determination of the stable metabolites: nitrates and nitrites using the Griess reaction. Blood was taken from the left hand finger on an empty stomach; the patient had previously followed a strict diet in order to prevent ingestion of sources of nitrates from food and smoking. The obtained sample was centrifuged for 10 minutes to obtain serum. Saliva was obtained from the right parotid saliva by placing a special cotton swab from a Salivette centrifuge tube from Sarstedt (Germany), soaked in citric acid, in the area of the Stenon's duct orifice, and asking the patient to thoroughly suck the swab. Saliva was collected at a specific time (08 : 58). Then, the saliva was also centrifuged. The resulting serum and centrifuged saliva were frozen and stored at −20°C in the laboratory. In the laboratory, samples were frozen and stored at *t*−20°C until measurement. Then, the samples were thawed (to room temperature) and deproteinized by adding 0.8 ml of 0.5 m NaOH and 0.8 ml of 10% zinc sulfate solution to 0.4 ml of the sample. After that, the protein content in the samples did not exceed 20–50 mg/*l* (Bradford's colorimetric method). The contents of the tube were mixed for 30 seconds and centrifuged for 15 minutes at 9000 rpm/min (10000 g). Cadmium granules (mass fraction of granular cadmium >99.96%) were added to the supernatant liquid to reduce NO_3_^−^ to NO_2_^−^. The cadmium granules were preliminarily washed with bidistilled water, with 1 m HCl, and again with bidistilled water until a neutral reaction of the medium was obtained. The parameters of completeness of the reduction of nitrate to nitrite by cadmium granules added to the samples were determined from the concentration dependence of nitrite, verified using the Griss reaction. The return of NO_3_^−^added to blood serum samples under the influence of cadmium granules was 96–105%. After that, an equal volume of Griss reagent (1% sulfanilamide, 0.1% naphthylenediamine, and 2.5% phosphoric acid) was added to the supernatant (1.5 ml) and incubated for 10 minutes at room temperature. The absorption of the solution was measured on a spectrometer at a wavelength of 546 nm. The NOx concentration was determined using a standard, which was sodium nitrite.

### 2.2. Statistical Data Processing Methods

The statistical processing of the study results included the using of student's *t*-test with a significance level (*p*) which was equal to 0.05 or 0.01 and the Pearsons parametric method for determining the relationship (correlation) between variables.

## 3. Results

**Figure 1:**
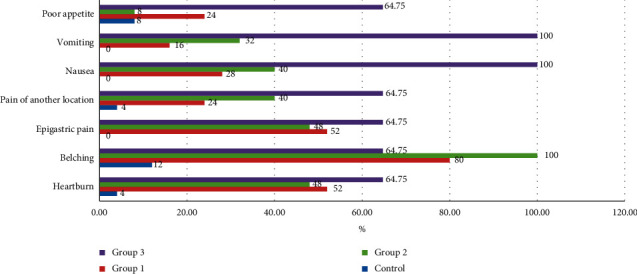
The comparative prevalence of esophageal complaints among patients with GERD with different types of refluctant.

The data analysis of the questionnaires of the GERD general clinical manifestations showed that the most common complaint among patients with the prevailing of ACR or SACR was belching (in 80.0% and 100.0% of cases, respectively) and among patients with the prevailing of SALCR, the nausea and single vomiting (in 100.0% of cases, respectively) (Figure 1).

**Figure 2:**
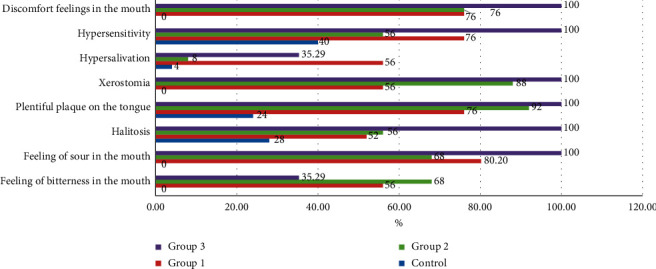
The comparative prevalence of dental complaints among patients with GERD with different types of refluctant.

The control group patients mainly had complaints of halitosis and the teeth hypersensitivity (28.0% and 40.0%, respectively). The most common complaints among patients with the prevailing of ACR were the complaints of the feeling of sour in the mouth (80.2%). The complaints of the plentiful plaque on the tongue and xerostomia were the main complaints among patients with the prevailing of SACR (92.0% and 88.0%, respectively). The complaints of feeling of sour in the mouth, halitosis, plentiful plaque on the tongue, xerostomia, hypersensitivity of teeth, and discomfort feeling in the mouth occurred more often among patients with the prevailing of SALCR (in 100.0% of cases) than in the other groups (Figure 2).

According to the objective dental examination among patients of the main groups (and in 100.0% of cases in Group 3), the swelling of the oral mucosa, plentiful plaque on the tongue, and hyposalivation prevailed (Table 1).

During the measuring of the functional parameters of saliva, the lowest value of the salivation rate was observed in Group 3 (0.10 ± 0.04 ml/min), and the salivation rates in Groups 1 and 2 were slightly higher than in Group 3 and were at the lower limit of the normal values (0.32 ± 0.19 ml/min). This fact can be described by the violation of the salivary-esophageal reflex (Table 2).

During the study of the buffer capacity of saliva among patients with GERD, some abnormalities were detected. The highest level of buffer capacity of saliva was observed in Group 2 (9.40 ± 1.71 mmol eq/l) (Table 2) and the lowest was observed among patients with the prevailing of SALCR (7.63 ± 0.18 mmol eq/l) (Table 2).

The tendency of increasing of the BC of saliva in Groups 1 and 2, apparently, may indicate the increasing of the protective role of saliva bicarbonate system to the aggressive nature of refluctant due to the neutralization of its contents. The protective role of bicarbonate system of saliva in Group 3 was low. This fact may indicate the decompensation of the mechanisms of the antireflux barrier in response to the more aggressive components of refluctant.

The highest level of NOx in blood serum was determined among patients with the prevailing of SACR (27.87 ± 1.14 umol/), and the lowest was among patients with the prevailing of SALCR (25.37 ± 2.55 umol/l). The highest level of NOx in SRPSG was identified among patients with the prevailing of SALCR (20.93 + 11.23 umol/l) and the lowest was among patients with the prevailing of ACR (14.57 + 5.16 umol/l) (Table 3).

**Figure 3:**
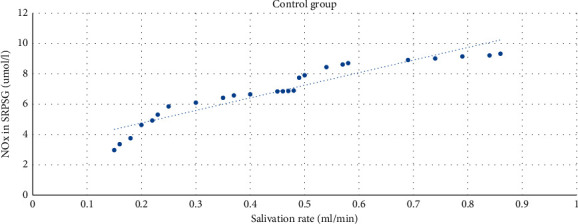
The correlation between the NOx level in SRPSG and the salivation rate among patients in the control group.

The strong direct correlation (*r* = 0.94) between the NOx level in SRPSG and the salivation rate was found among healthy individuals. The correlation was significant at the level of 0.01 (bilateral) (Figure 3).

**Figure 4:**
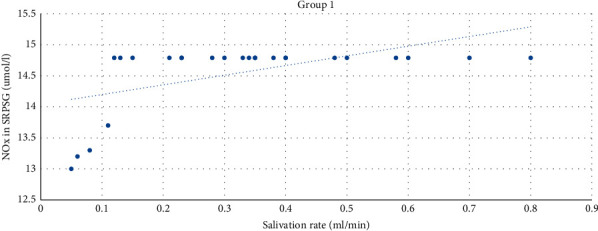
The correlation between the NOx level in SRPSG and the salivation rate among patients in Group 1.

The moderate direct correlation between the NOx in SRPSG and the salivation rate (*r* = 0.55) among patients with the prevailing of ACR was revealed. The correlation was significant at the level of 0.01 (bilateral) (Figure 4).

**Figure 5:**
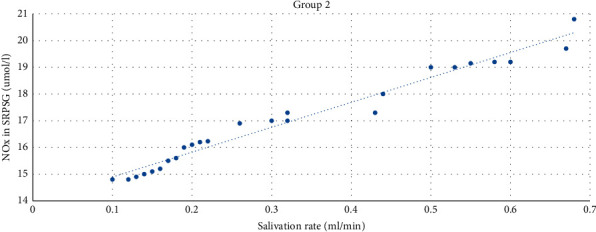
The correlation between the NOx level in SRPSG and the salivation rate among patients in Group 2.

The strong direct correlation (*r* = 0.98) between the level of NOx in SRPSG and the salivation rate was revealed among patients with the prevailing of SACR. The correlation was significant at the level of 0.01 (bilateral) (Figure 5).

**Figure 6:**
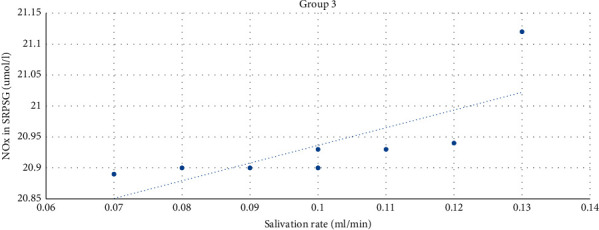
The correlation between the NOx level in SRPSG and the salivation rate among patients in Group 3.

The strong direct correlation between the level of NOx in SRPSG and the salivation rate (*r* = 0.75) was found among patients with the prevailing of SALCR. The correlation was significant at the level of 0.01 (bilateral) (Figure 6).

**Figure 7:**
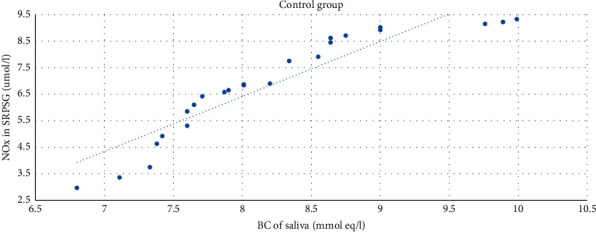
The correlation between the NOx level in SRPSG and the buffer capacity of saliva among patients in the control group.

The strong direct correlation between the BC of saliva and the NOx level in SRPSG among patients in the control group was revealed (*r* = 0.92; *p* < 0.01 (bilateral)) (Figure 7).

**Figure 8:**
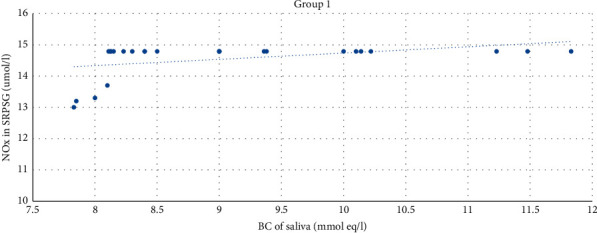
The correlation between the NOx level in SRPSG and the buffer capacity of saliva among patients in Group 1.

The moderate direct correlation was also revealed between the level of buffer capacity of saliva and the NOx level in SRPSG among patients in Group 1 (*r* = 0.42). The correlation was significant at 0.05 (bilateral) (Figure 8).

**Figure 9:**
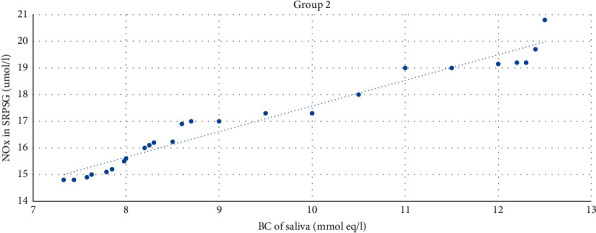
The correlation between the NOx level in SRPSG and the buffer capacity of saliva among patients in Group 2.

The strong direct correlation between the NOx level in SRPSG and the buffer capacity of saliva was revealed among patients in Group 2 (*r* = 0.97; *p* < 0.01) (bilateral) (Figure 9).

**Figure 10:**
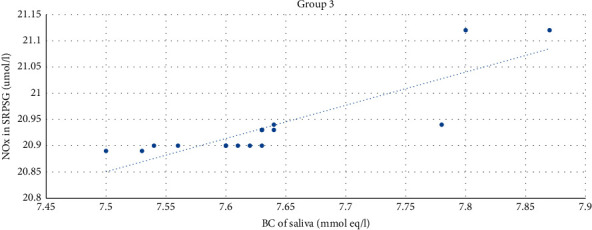
The correlation between the NOx level in SRPSG and the buffer capacity of saliva among patients in Group 3.

The strong direct correlation between the level of BC of saliva and the NOx level in SRPSG was established among patients in Group 3 (*r* = 0.86). The correlation was significant at the level of 0.01 (bilateral) (Figure 10).

## 4. Discussion

The leading number of complaints and dental manifestations of GERD was observed among patients with the prevailing of SALCR. The salivation rate among patients with GERD with the prevailing of ACR and SACR was at the lower limit of normal values (0.32 ± 0.19 ml/min), and the salivation rate among patients with the prevailing of SALCR was low (0.10 ± 0.04 ml/min). The BC of saliva among patients with the prevailing of ACR and SACR was high (9.07 ± 1.23 mmol eq/l and 9.40 + 1.71 mmol eq/l, respectively), and was reduced among patients with the prevailing of SALCR (7.63 ± 0.18 mmol eq/l). The NOx level in SRPSG among patients with GERD was increased (especially in Group 3 (20.93 ± 11.23 umol/l)).

Thus, the worst situation of the oral cavity state was observed among patients with GERD with the prevailing of SALCR probably due to the more aggressive nature of refluctant. Accordingly, during the process of revealing the manifestations of GERD in the oral cavity, the following additional diagnostic methods can be used: the sialometry and the measurement of the BC of saliva.

The differences in the subjective condition, the level of nitric oxide metabolites in saliva from the right parotid salivary gland, the buffer capacity of saliva, and the salivation rate were found; the correlation between the parameters of saliva and the prevailing character of refluctant (acidic, slightly acidic, and slightly alkaline) was defined among patients with gastroesophageal reflux disease [1, 3–7].

The higher the level of nitrite/nitrate in the saliva, the less it is in the serum and vice versa. It can be assumed that this phenomenon may be based on profound changes in the system of nitrate formation and NO deposition in the body. In addition to the exogenous origin of nitrates (with water, food, and tobacco smoke), the supply of nitrates to the salivary glands from the blood (nitrates are absorbed in the small intestine, after which 25% of the nitrates entering the body accumulates in the salivary glands), there is also an endogenous origin of nitrates (NOS pathway: the process of NO formation from L-arginine in the presence of О_2_ directly in the tissues of the oral cavity under the influence of nitroxide synthase enzymes with the subsequent conversion of NO as a result of its interaction with molecular О_2_ into NO_3_^−^ and NO_2_^−^ ions) [2, 8, 9].

The secretion of the salivary glands contains such protective factors as bicarbonates, epithelial growth factor, and prostaglandin E2, which transform the growth factor. They are deposited in the mucous membrane of the esophagus and form a buffer layer in which a neutral pH is maintained, thus performing the function of an “umbrella” under the aggressive action of refluctate [1]. Our studies have shown that the higher the rate of salivation in patients with GERD, the higher the content of NO metabolites in the LSS, and conversely, the lower the level of NOx in the LSS, the lower the rate of salivation.

The concentration of NOX increases in patients with GERD and the control group in direct proportion to the increase in the rate of salivation. This phenomenon can also be explained by an increase in the adaptive mechanisms of biological systems by an increase in the content of NO in LSS due to an increase in its synthesis (L-arginine-NO pathophysiological path; anion-nitrates; nitrate-reductase pathway) or the release of nitric oxide from the depot, for example, from cells of the right parotid salivary gland.

If the salivation rate among patients with GERD is high, respectively, the NOx level in SRPSG is high too. However, if the NOx level in SRPSG is low, the salivation rate is low too. This fact can be described, according to Casselbrant et al. [1], by the remote exposure of NO which can increase the salivary glands secretion and the formation of the protective buffer layer in the esophageal mucosa against the aggressive action of reflux [3].

According to our research results, it can also be argued that the higher the level of BC in the oral fluid in patients with GERD, the higher the level of nitrates/nitrites in the SRPSG, and conversely, the lower the level of NOx in the SRPSG, the more the level of the buffer capacity of the mixed saliva is lower.

Thus, when the saliva BC level is high, the NOx level in SRPSG is directly proportionally high too, possibly due to the L-arginine-NO pathophysiological pathway [10]. The increasing of the BC level of saliva among patients with GERD may be adaptive in response of reaching the aggressive contents of the stomach or the stomach and duodenum or the oral cavity.

## 5. Conclusion

The worst situation of the oral cavity state was observed among patients with GERD with the prevailing of SALCR, probably due to the more aggressive nature of refluctant. Accordingly, during the process of revealing the manifestations of GERD in the oral cavity, the following additional diagnostic methods can be used: the sialometry and the measurement of the BC of saliva.

The direct correlation between the indicators of sialometry, the level of the BC of saliva, and the NOx level in SRPSG were established during the study.

According to the data obtained, the method of determining the NOx level in SRPSG can be recommended to be used as the additional method of the differential diagnosis among patients with GERD with different types of refluctant in dentistry and gastroenterology.

## Figures and Tables

**Table 1:** The oral examination results among patients with GERD.

Groups	Control (*n* = 25)		Group 1 (*n* = 25)		Group 2 (*n* = 25)		Group 3 (*n* = 17)	
Symptoms	*n*	%	*n*	%	*n*	%	*n*	%
Hyposalivation	7	28.0	16	64.0	14	56.0	17	100
Moderate salivation	16	64.0	8	32.0	8	32.0	0	0
Hypersalivation	2	8.0	1	4.0	2	8.0	0	0
Plaque on the tongue	18	72.0	19	76.0	19	76.0	17	100
Swelling of the oral mucosa	12	48.0	18	72.0	21	84.0	17	100
Hyperkeratosis symptoms of the oral mucosa	0	0	1	4.0	0	0	11	64.7
Atrophy of the tongue papillae	2	8.0	3	12.0	6	24.0	0	0

**Table 2:** The functional parameters of mixed saliva in patients with GERD.

Groups	Sialometry (ml/min)	BC of saliva (mmol eq/l)
Control (*n* = 25)	0.45 ± 0.16	8.20 ± 0.84
Group 1 (*n* = 25)	0.32 ± 0.19	9.07 ± 1.23
Group 2 (*n* = 25)	0.32 ± 0.19	9.40 ± 1.71
Group 3 (*n* = 17)	0.10 ± 0.04	7.63 ± 0.18^†^
Total (Groups 1, 2, and 3) (*n* = 67)	0.30 ± 0.15	8.99 ± 1.39^†^

^*∗*^the differences with the control group are statistically significant in all groups (*p* ≤ 0,05 − 0,01). ^†^*p* > 0.05, the results are not reliable when compared to the control group.

**Table 3:** Patients distribution according to the results of NOx determination in SRPSG.

Groups	NOx in SRPSG (*μ*mol/l)
Control (*n* = 25)	6.84 ± 2.55
Group 1 (*n* = 25)	14.57 ± 5.16
Group 2 (*n* = 25)	16.98 ± 4.18
Group 3 (*n* = 17)	20.93 ± 11.23
Total (Groups 1, 2, 3) (*n* = 67)	15.95 ± 7.75

‡, the differences with the control group are statistically significant in all groups (*p* ≤ 0.01).

## Data Availability

The underlying data used in this clinical study are available upon request sent to G. I. Lukina via lukinagi@mail.ru.
